# Human Lower Limb Motion Capture and Recognition Based on Smartphones

**DOI:** 10.3390/s22145273

**Published:** 2022-07-14

**Authors:** Lin-Tao Duan, Michael Lawo, Zhi-Guo Wang, Hai-Ying Wang

**Affiliations:** 1 School of Information and Software Engineering, University of Electronic Science and Technology of China, Chengdu 610054, China; duanlintao@cdu.edu.cn; 2 School of Computer Science, Chengdu University, Chengdu 610106, China; wanghaiying@cdu.edu.cn; 3 International Graduate School for Dynamics in Logistics, Bremen University, 28359 Bremen, Germany; mlawo@tzi.de

**Keywords:** human motion recognition, motion sensor, smartphone, supervised learning algorithms

## Abstract

Human motion recognition based on wearable devices plays a vital role in pervasive computing. Smartphones have built-in motion sensors that measure the motion of the device with high precision. In this paper, we propose a human lower limb motion capture and recognition approach based on a Smartphone. We design a motion logger to record five categories of limb activities (standing up, sitting down, walking, going upstairs, and going downstairs) using two motion sensors (tri-axial accelerometer, tri-axial gyroscope). We extract the motion features and select a subset of features as a feature vector from the frequency domain of the sensing data using Fast Fourier Transform (FFT). We classify and predict human lower limb motion using three supervised learning algorithms: Naïve Bayes (NB), K-Nearest Neighbor (KNN), and Artificial Neural Networks (ANNs). We use 670 lower limb motion samples to train and verify these classifiers using the 10-folder cross-validation technique. Finally, we design and implement a live detection system to validate our motion detection approach. The experimental results show that our low-cost approach can recognize human lower limb activities with acceptable accuracy. On average, the recognition rate of NB, KNN, and ANNs are 97.01%, 96.12%, and 98.21%, respectively.

## 1. Introduction

Modern Smartphones have become increasingly popular in people’s daily life due to their rich context-aware supporting applications beyond basic voice service capabilities [1,2,3]. Human motion recognition is one of these applications; it provides interpretations of the sensed data of human activities for a good user experience.

Human motion recognition has been the target of extensive studies using either external or wearable sensors [4]. External sensors like cameras at fixed locations focus on human activity by capturing human motion. Wearable motion capture systems include several inertial measurement units (IMUs) fixed on different parts of the user: head, shoulders, chest, arms, wrists, palms, fingers, hip, upper legs, lower legs, and feet. The IMUs measure the accelerations and angular velocities of different body parts in real time. Where, with external sensors, motion outside the field-of-view is invisible and requires multiple cameras for motion capturing in larger areas, wearable motion capturing is obtrusive and inconvenient when one has to connect by wire several IMUs on different parts of the body. In addition, both systems are not free of cost. 

Fortunately, modern smartphones are equipped with a variety of sensors, such as an accelerometer, gyroscope, magnetometer, and camera; it has become feasible to develop human motion monitoring and recognition algorithms using one or more of these sensors on smartphones without any inconvenience. Thus, we propose in this paper human lower limb motion capturing and recognition using the IMU built into an Android-based Smartphone. The IMU measures unobtrusively the nine values of the tri-axial accelerometer, gyroscope, and magnetometer at no extra cost. We investigate the features of sensing data and recognize five daily activities of users using three supervised learning algorithms with an acceptable accuracy. In this work, we aim to detect in real time subjects’ five daily physical activities by training classifiers using sensing data received from motion sensors of their own Smartphones. Our contributions mainly include four aspects:

1. We design a data logger to collect human lower limb motion data in real time using the embedded accelerometer and gyroscope of an Android-based Smartphone;
2. In order to reduce the number of dimensions without degrading the recognition rate, we conduct several experiments to find the optimal subset of features vector using the K-folder cross-validation technique, because the large number of dimensions of the feature vector may induce the complexity of the classifier model and increase need for system resources, such as computing, storage, and energy;
3. With the trained models, one can recognize five human lower limb activities with a high recognition rate. According to our experiments, we found sitting down is easiest identified among the five human lower limb motions, whatever classifier one applies. The Artificial Neural Network classifier has the best recognition performance, whereas the Naïve Bayes classifier has the best recognition rate for going downstairs and the worst recognition rate for the walking activity;
4. We implement an activity live detection program for Android-based Smartphones using our proposed human lower limb motion capture and recognition method.

The organization of the rest of this paper is as follows: In Section 2, we analyze the state-of-the-art from related works. Section 3 proposes our approach. In Section 4, we present our experimental results and evaluate our motion capture and recognition approach. Finally, this paper draws conclusions and outlines future work in Section 5.

## 2. Related Work

External and wearable sensors can capture human motion [5,6,7]. External sensors are able to gather complex human activities. Nguyen et al. [8] placed motion capture suits equipped with 17 IMUs positioned on each limb, trunk, and head segment of participants to monitor full-body 3D movement, such as sitting down, standing up, reaching, walking, and segmentation. Nguyen et al. employed nonlinear transform and adaptive thresholds to detect peaks that correspond to different activities. Wang et al. [9] used cameras to record human motion and studied first-person daily activity recognition from video streams utilizing object hypotheses and deep convolutional neural network–based detection frameworks. Hamdi et al. [10] connected seven IMUs by wire and fixed them on the body’s waist and lower limb segments. Each IMU was composed of a tri-axial gyroscope and a tri-axial accelerometer able to measure the subject’s motion along three orthogonal axes. Chinimilli et al. [11] placed one inertial measurement unit on a person’s thigh to capture the thigh angular data while the subject was moving. 

In recent years, one also finds research on motion capturing with Smartphone sensors. Filios et al. [12] proposed a hybrid recognition model to detect four daily human activities using only one tri-axial accelerometer sensor of a Smartphone. This approach has high accuracy and is convenient as it does not disturb people’s daily life. Anjum et al. [13] recognized human physical activities on a Smartphone in real time; their mobile application monitored seven different human activities without space and time limitations. Belman et al. [14] collected walking, upstairs, and downstairs motion data from 117 subjects using both Samsung-S6 and HTC-One mobile phones. They shared their dataset as a public dataset named Syracuse University and Assured Information Security-Behavioral Biometrics Multi-Device and Multi-Activity Data (SU-AIS BB-MAS), which is hosted by the IEEE-Dataport [15]. The dataset consists of 57.1 million data-points for both accelerometers and gyroscopes and provides a better context for human activity recognition.

Apart from obtaining the sensing data of human motion, feature extraction and classifier selection are also important steps in the process of human motion recognition. As for motion features, most literature, such as Refs. [10,11,12,16], extract features from time series of sensing data. Others extract motion features from the frequency domain of raw data [17,18]. As for classifiers, machine learning algorithms are widely used, such as K-nearest neighbor, support vector machine, naïve Bayes, neural networks, Markov models, and convolutional neural networks [9,11,13,19,20]. No matter which one uses, one first needs the motion features to extract from the raw sensing data. The feature selection directly affects the performance and energy consumption of the recognition system. We use frequency, magnitude, and the phase as motion features extracted in the frequency domain of sensing data for the FFT method.

Hamdi et al. [10] found that the motion features from one leg are sufficient for lower limb motion recognition because both legs have the same profile. Therefore, in order to obtain the human daily activities with high accuracy at low cost, we fixed one Smartphone on the right upper leg and used the tri-axial accelerometer and tri-axial gyroscope to collect back and forth motion data in the sagittal plane [12]. We extracted motion features in the frequency domain of sensing data using FFT to detect lower limb motion offline using three supervised learning algorithms on a high performance computer, as the data processing requires hardware resources and costs battery energy. Although Gabor Wavelet Transform is also an effective time-frequency analysis method, the Fast Fourier Transform is suitable for processing long sequential stable signals like the human lower limb motion in this study. The FFT is a computationally fast and efficient way to implement the Discrete Fourier Transform (DFT), which is a linear transformation that extracts the frequency content of a vector or a discrete signal [21]. It is formulated as follows:

(1) $$X(k)=\sum_{n=0}^{N-1}x(n)(\cos\frac{2\pi nk}{N}-i\sin\frac{2\pi nk}{N}),k=0,1,2\ldots ,N-1,$$

where *N* is the length of the signal/vector and *X*(*k*) is the content at the frequency of 2*πk*/*N*.

## 3. Method

In this work, we implement a low-cost motion recognition system using only the accelerometer and gyroscope of a Smartphone widely used in daily life. By fusing the sensing data of the two sensors, we achieve a more effective recognition than with only the tri-axial accelerometer.

### 3.1. System Architecture

**Figure 1:**
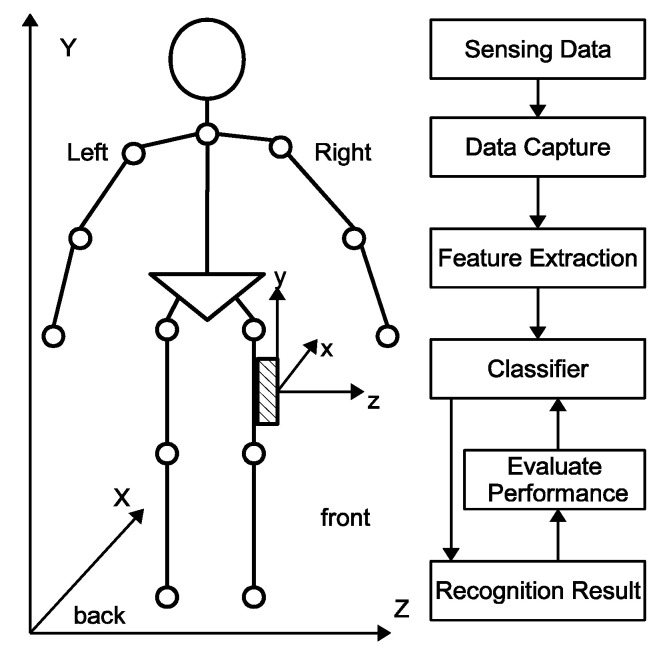
Overview of the proposed system architecture.

Figure 1 shows on the right the overall system architecture for human lower limb motion capture and recognition. The three main building blocks are:

1. Motion Data Capturing: The Smartphone has an Inertial Measuring Unit (IMU) with three built-in tri-axial sensors: accelerometer, gyroscope, and magnetometer. We attach the Smartphone on the right thigh. We record on a SD Card for later upload to a remote server and off-line processing of the three accelerations measured by the accelerometer and the three angular velocities measured by the gyroscope.
2. Feature Extraction: FFT is a useful mathematical tool in signal processing, as it transforms data from the time into the frequency domain. We use the frequencies, magnitude, and phase within the frequency domain as motion features. Based on a large number of experiments, we could reduce with little effect on the probability of information loss the dimension of the features vector.
3. Classifier: We use three classifiers: NB, KNN, and ANN. We train and evaluate these classifiers using the 10-fold cross-validation method, with 90% of data for training and 10% for testing.

### 3.2. Motion Data Collection

For the data collection, we fixed the Smartphone as mentioned on the end user’s right thigh. The embedded motion sensor uses a right-hand spatial coordinate system calibrated when the user stands upright (see Figure 1). In this position, the *x*-axis points straight ahead, the *y*-axis points above against gravity, and the *z*-axis points right related to the global X-Y-Z coordinate system.

The sensors in Android-based Smartphones provide four kinds of sampling rates *f_sample_*: FASTEST (≥50 Hz, which depends on the user’s platform), GAME (50 Hz), UI (16 Hz), and NORMAL (5 Hz) [12,13]. We set the sampling rate to NORMAL for two reasons:

1. Android systems typically use a smaller sampling rate, which is adequate for a human’s normal activity in their daily life [13];
2. Smartphones use the least battery energy with this sampling rate because of a lower load on the processor.

However, it is worth noting that we set the sampling rate to UI (16 instances per second) in our live detection system for achieving more detailed motion sensing data, because the human’s fast activities need a much higher sampling frequency based on the Nyquist’s sampling theorem, which states that the signal rate should not be higher than half the sampling rate.

To investigate the lower limb motion in the sagittal plane, we only collect three values of the linear accelerometer (excluding gravity) and calibrated gyroscope: the acceleration along the *x*-axis (*A_x_*) and along the *y*-axis (*A_y_*) as well as the angular velocity around the *z*-axis (*G_z_*).

Keeping the variety of motion and improving the reliability, we take under different conditions 670 samples of five activities (sitting down, standing up, walking, going upstairs, and going downstairs). We vary the stride length of walking, the step height of stairs, and the height of chairs.

**Figure 2:**
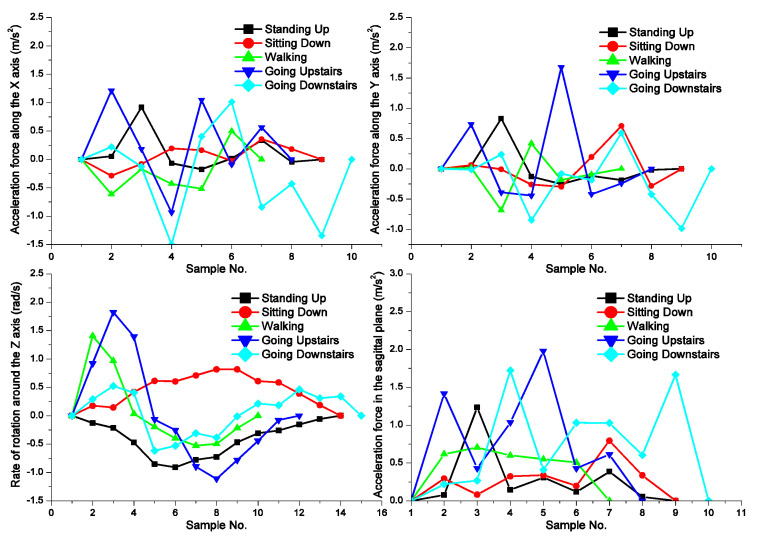
Sensed accelerations and velocity of each gait cycle.

Figure 2 shows the time series of the three values of interest (*A_x_*, *A_y_*, and *G_z_*) for each gait cycle of the five activities. The acceleration in the sagittal plane (*A_sag_*) in Figure 2 results from the accelerations *A_x_* and *A_y_*, as follows:

(2) $$A_{sag}=\sqrt{A_{x}{}^{2}+A_{y}{}^{2}}.$$

### 3.3. Feature Extraction

**Figure 3:**
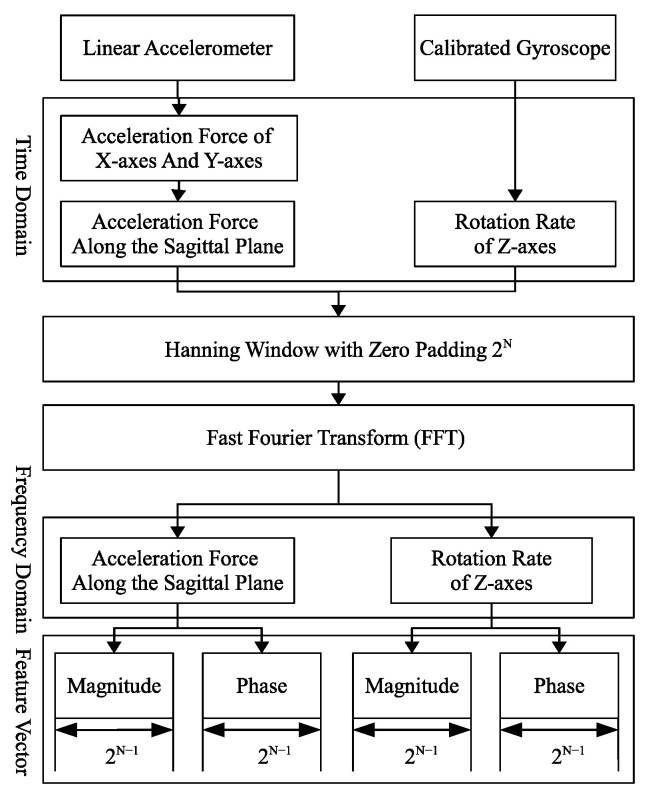
The process of feature extraction.

One needs to calibrate the raw data of sensors because of noise, gravity, and gyro-drift. The Smartphone sensors provide raw sensing data from the accelerometer and uncalibrated gyroscope. In this paper, we use calibrated sensing data according to the linear accelerometer (excluding gravity) and gyroscope (including gyro-drift compensation). In an Android-based Smartphones, Android platforms support several motion sensor types to collect gait sensing data. Among them, the acceleration sensor (TYPE_ACCELEROMETER) measures the acceleration force along each of the three axes including gravity, the linear acceleration sensor (TYPE_LINEAR_ACCELERATION) collects a three-dimensional vector indicating acceleration along each device axis, not including gravity, the gyroscope sensor (TYPE_GYROSCOPE) measures the rate of rotation around the device’s *X*, *Y* and *Z* axis, and the uncalibrated gyroscope sensor (TYPE_GYROSCOPE_UNCALIBRATED) provides the rate of rotation around the device but no gyro-drift compensation is performed. Fortunately, consumers can use gyro-drift bias values to calibrate the given sensor values. In this study, we use TYPE_LINEAR_ACCELERATION and TYPE_GYROSCOPE to collect the calibrated sensing data. Figure 3 shows the block diagram of the feature extraction. We use the Hann function as a window function and pad with zero to extend the length of the signal to 2*^N^* (*N* = 1, 2, 3, …) for the FFT. We obtain four values: the magnitude of frequency domain of *A_sag_* (*M_sag_*), the phase of frequency domain of *A_sag_* (*P_sag_*), the magnitude of frequency domain of *G_z_* (*M_z_*), and the phase of frequency domain of *G_z_* (*P_z_*), as follows:

(3) $$M_{sag}=\mathrm{abs}(\mathrm{FFT}([\mathrm{hanning}(A_{sag}),\mathrm{zeros}(1,2^{N}-\mathrm{length}(A_{sag}))])),$$

(4) $$M_{z}=\mathrm{abs}(\mathrm{FFT}([\mathrm{hanning}(G_{z}),\mathrm{zeros}(1,2^{N}-\mathrm{length}(G_{z}))])),$$

(5) $$P_{sag}=\mathrm{angle}(\mathrm{FFT}([\mathrm{hanning}(A_{sag}),\mathrm{zeros}(1,2^{N}-\mathrm{length}(A_{sag}))])),$$

(6) $$P_{z}=\mathrm{angle}(\mathrm{FFT}([hanning(G_{z}),\mathrm{zeros}(1,2^{N}-\mathrm{length}(G_{z}))])).$$

where abs, angle, FFT, and hanning are the magnitude, phase, Fast Fourier Transform, and hanning window operation used in Matlab, respectively. In this paper, *N* is set to be 5. Finally, we obtain the feature vector X = {*M_sag_*, *P_sag_*, *M_z_*, *P_z_*}. The length of *M_sag_*, *P_sag_*, *M_z_*, and *P_z_* is 2*^N^*^−1^ due to the symmetry. Therefore, the original integrated motion feature vector includes 2*^N^*^+1^ elements.

However, the large number of dimensions of the feature vector may induce the complexity of the classifier model and increase the need for system resources (computing, storage, and energy). To reduce the number of dimensions of the motion features vector, we take the first *n* order features from each magnitude and phase vector to form a subset of motion features as a new feature vector with 4 × *n* elements. The subset of the motion feature vector is thus as follows:

(7) $$X=\{M_{sag}(1\ldots n),P_{sag}(1\ldots n),M_{z}(1\ldots n),P_{z}(1\ldots n)\},n=1,2,3,\ldots ,2^{N-1}.$$

### 3.4. Classifier

Human activity recognition is an active and challenging research area due to its applications in different areas like healthcare and security. A large portion of works related to this center around breaking down the execution of grouping calculations using different machine learning algorithms like Naive Bayes, Multi-Layer Perceptron, and K-Nearest Neighbors. The three classifiers’ characteristics are shown as follows. Naive Bayes classifier treats all features as independent and is by far the simplest of the three classifiers. K-Nearest Neighbors is a machine learning model, and this algorithm shows the characteristics of instance-based learning. It is mostly used as a method of classification in which grouping of examples is dependent on their coordinates and distance from others in the feature space. The Artificial Neural Network classification method has a multi-layer architecture, including input, hidden, and output layers. The nodes in adjacent layers fully connect from a lower layer to a higher layer. These three algorithms have been used successfully in activity recognition with a varying success rate.

We use three classification methods to recognize human lower limb motion, including Naïve Bayes, K-Nearest Neighbors, and Artificial Neural Networks.

With the Naïve Bayes classifier, one can assume that features of classes are independent. The Naïve Bayes classifier is

(8) $$h(X)=\arg\underset{i}{\max}\prod_{j=1}^{m}P(x_{j}|C=i)\cdot P(C=i).$$

where *X* = {*x*_1_, *x*_2_, …, *x_m_*} is the feature vector, including m attributes of a given sample x, and *C* ∈ {1, 2, …, numLabels} is the class label of the feature vector *X*. *P*(*X* = *x_j_*|*C* = *i*) is the conditional probability when class *C* equals *i*. P(*C* = *i*) is the prior probability of *C* equaling *i*. The given example *x* belongs to class *i* with the maximum a posteriori probability hypothesis *h*(*x*). In this paper, we filter all the features in which the variance of training and testing dataset is not positive.

The K-Nearest Neighbors algorithm is based on the Euclidean distance between a test sample and the specified training samples. The test sample *x* belongs to the majority class label of its K nearest training samples. In this paper, we set K equal to 1. The class label of sample *x* is the same as the class label of the nearest neighbor *x_traini_* with the minimum Euclidean distance d:

(9) $$\arg\underset{i}{\min}d(x,x_{traini}).$$

Like the two learning algorithms mentioned above, the Artificial Neural Network (ANN) classification method has a multi-layer architecture, including input, hidden, and output layers. The nodes in adjacent layers fully connect from a lower layer to a higher layer. In this paper, we use ANN based on the feed-forward backpropagation algorithm and set the number of input nodes to be 4 × *n*, which is equal to the size of the feature vector. We select the optimal number of hidden nodes using a trial and error approach for the cross-validation procedure. We set the number of output nodes to be numLabels = 5 because the number of class labels is five, corresponding to five different lower limb activities.

In order to evaluate the three algorithms, we use a 10-fold cross-validation technique, also used in [11,13]. We divide the samples equally into ten portions. We use one portion as test dataset and the other nine portions as training datasets. The second time, we select another unselected portion as the test set and the remaining nine portions as training sets. We can obtain a recognition accuracy for each training and testing process. After ten steps, the average accuracy is the recognition rate of the classifier.

### 3.5. Live Detection Algorithm

Our objective is to find an algorithm for mobile users in order to recognize the human lower limb motion in real time such that the proposed motion recognition method may be applied in a real scenario. In order to solve the problem, we suggest a live detection algorithm for human lower limb motion based on our motion recognition method.

The summary of the flow of the live detection algorithm is shown as follows. At first, we achieve the total number of sample points and the maximum number of sample points for each motion cycle based on the sample frequency *f_sample_* and the length of time serial *t_acc_*, *t_gyr_* (lines 1–2). Then, we find all the peak locations of time series from the start location to the maximum position of an activity cycle (line 4). For each peak location, we find the forward cross zero points as the end location and store them in an end location vector (line 5). After that, we extract the features of human activity from the time series fragment of accelerometer and gyroscope between start location and end location using our FFT feature extraction method (lines 6–8). Finally, we use the ANN classifier with the best recognition performance to predict the human activity (line 9). The activity is the prediction with the maximum probability that is no less than the acceptable accuracy (lines 10–12). The Algorithm 1 takes O(M × logN) time for the feature extraction phase by FFT time-frequency transform in step 8 and O(M × numHLN) time for the motion prediction phase by ANN in step 9. The Algorithm 1 has a O(M × logN + M × numHLN) time complexity for M sampling points, N sampling points of each motion cycle and numHLN hidden layer nodes of ANN.
**Algorithm 1.** Human Lower Limb Motion Live Detection Algorithm**INPUT**: time series of accelerometer ***t****_acc;_* time series of gyroscope ***t****_gyr_* **OUTPUT**: ***activities*** 1: *numTotalSample* ← *f_sample_* × the length of time series***t**_acc_*, ***t****_gyr_* 2:* numMaxCycleSample* ← *f_sample_* × *periodMaxCycle* 3: **WHILE** *startLocation* < *numTotalSample*
**DO** 4:  ***locs*** ← findPeaks(*startLocation*: *startLocation* + *numMaxCycleSample*) 5:  ***endLocation*** ← searchEndLocation(***t****_acc_*, ***t****_gyr_*, ***locs***) 6:  ***acc*** ← ***t****_acc_*(*startLocation*: ***endLocation***) 7:  ***gyr*** ← ***t****_gyr_*(*startLocation*: ***endLocation***) 8:  ***x*** ← featureExtractionByFFT(***acc***, ***gyr***) 9:  ***prob***, ***pred*** ← ANN(***x***) 10:   *prob_optimal_* ← maximum(***prob***) 11:   **IF** *prob_optimal_* ≥ ACCEPTABLE_ACCURACY **THEN** 12:   ***activities*** ← maximum(***pred***) 13:   *startLocation* ← ***endLocation***(*prob_optimal_*); 14:   **ELSE** 15:   *numEndLoc* ← length of ***endLocation*** 16:   *startLocation* ← ***endLocation***(*numEndLoc*-1) 17:   **END IF** 18: **END WHILE**

## 4. Results

### 4.1. Preparation of Data Set

To reduce the degree of limitation, we only use two motion sensors: a linear accelerometer (range: 19.61 m/s^2^, resolution: 5.98 × 10^−4^ m/s^2^, vendor: Google Inc. (Mountain View, CA, USA) and a gyroscope (range: 8.72 rad/s, resolution: 2.66 × 10^−4^ rad/s, vendor: InvenSense^®^). Android versions 2.3 and later support these two sensors. In our experiments, we develop an Android application of data collection and deploy it on a Samsung Galaxy Note III Smartphone fixed on the right thigh of the end user. We set the accelerometer and gyroscope to work at a fixed sampling rate similar to the literature [22] and collect sensing data for each gait cycle recorded on an external storage. The sensing data consist of three time series of two motion sensors in the sagittal plane, represented as {*A_x_*, *A_y_*, *G_z_*}.

To increase the diversity of motion samples, we collected 670 examples under different conditions from eight international students whose ages range from 23 to 37 and heights range from 160 cm to 182 cm. Table 1 shows the variety of scenarios for five lower limb motions with variations. The stride length was between 50 and 90 cm during walking motion capturing. For going upstairs/downstairs, step heights were 17 and 20 cm. The height of chairs used was 32, 42, and 48 cm for sitting and standing motions. Table 1 also shows the percentage of samples for each activity (walking, going upstairs, going downstairs, standing up, and sitting down) is 23%, 18%, 17%, 21%, and 21%, respectively.

### 4.2. Determining Feature Vector Subsets

To determine the size of the motion feature vector, we conducted 320 times 10-fold cross-validation experiments for n first order feature set to 1 to 16 while the number of hidden layer nodes (numHLN) varied between 1 and 20.

**Figure 4:**
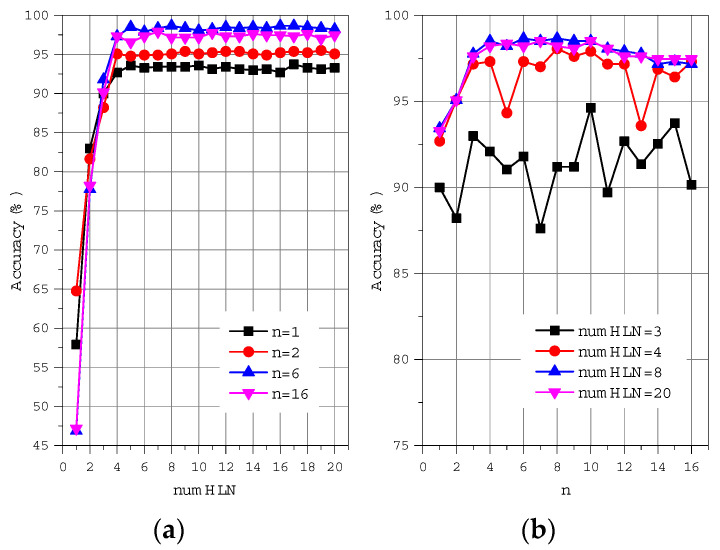
The impact of numHLN and n on recognition accuracy. (**a**) Accuracy varies with numHLN, (**b**) Accuracy varies with first order n.

Figure 4 shows the recognition accuracy, the number of hidden layer nodes, and the first n order of features. We calculated the average accuracy of each order and observed that we can trade off the number of motion features and recognition rate of lower limb motion when n is set to 6. As a result, we form a subset of features vector with 4 × 6 = 24 elements as a new feature vector for a better performance. These 24 features come from magnitude and phase of frequency domain, which is mentioned in the feature extraction section. Furthermore, we also tested different numbers for hidden layer nodes for ANN classifiers. Figure 4a shows the convergence of the recognition accuracy for a specific value *n* when numHLN is larger than 4. Figure 4b shows that the 10-fold cross-validation technique produces the best experimental results when the number of hidden layer nodes numHLN is set to 8 and the first order of features n is set to 6.

### 4.3. Classification Results

To evaluate our recognition method, we use five performance metrics: recognition accuracy, true positive rate (TPR), false positive rate (FPR), precision, and recall. Using the 10-fold cross-validation technique, we calculate these performance metrics. Table 2 shows the five human lower limb activities’ recognition results of the three classifiers. The first order of feature n for each classifier is set to be 6. That means we only use 24 features from the frequency domain of the sensed data for KNN and ANNs and use 22 features for NB because the within-class variance in two features of training is not positive.

From the experimental results, we can see that our human lower limb motion recognition method has high accuracy. ANNs with six hidden layer nodes have the best recognition performance, better than NB and KNN. On average, NB, KNN, and ANNs have 97.01%, 96.12%, and 98.21% recognition rates, respectively. Standing up and sitting down are easier to detect than the other three activities.

To sort errors of the three classifiers, we use the confusion matrix of Table 3. We use the notations L1, L2, L3, L4, and L5 to denote standing up, sitting down, walking, going upstairs, and going downstairs, respectively. Table 3 gives us three findings:

1. The Naïve Bayes classifier has the best recognition rate for going downstairs and the worst recognition rate for the walking activity;
2. Sitting down is the most easily identified among the five human lower limb motions, whatever classifier one applies. However, classification of walking, going downstairs, and going upstairs is sometimes incorrect;
3. The Artificial Neural Network classifier has the best recognition performance, although it has the most errors for going downstairs.

Furthermore, we conduct an extensive experiment using the public dataset SU-AIS BB-MAS to further demonstrate the effectiveness of our lower limb motion recognition approach. SU-AIS BB-MAS includes accelerometer and gyroscope raw data collected by inertial sensors built into smartphones. These activity-related raw data are logged on a mobile device when 117 participants with a smartphone in their pocket perform twice the following activities in sequence over five minutes: walking, going downstairs, walking, turning around, walking, going upstairs, and walking.

First of all, we perform a comprehensive analysis of the raw data of each participant using a time window of 1 s and find that the *z*-axis of a smartphone in a pocket does not always point right, as shown in Figure 1. We select the raw gait data of 10 users (participants 1, 6, 20, 40, 64, 66, 91, 101, 110, and 117) from SU-AIS BB-MAS and extract 754 samples. The reason why only 10 out of 117 users of SU-AIS BB-MAS are selected is that the placed position of the 10 users’ smartphone in their pocket is approximately similar to our approach, such that the *x*-axis of the smartphone points straight ahead, the *y*-axis points above, and the *z*-axis points right, related to the global X-Y-Z coordinate system. The percentage of samples for walking, going upstairs, and going downstairs is 53%, 25%, and 22%, respectively. We still use 10-folder cross-validation technique to calculate performance metrics. Table 4 shows the three activities’ recognition results of the three classifiers.

From the experimental results, we also can see that our human lower limb motion recognition method has high accuracy. ANNs with 64 hidden layer nodes has the best recognition performance, better than NB and KNN. All FPRs of ANNs are below 2%, with all other rates ranging between 93% and 100%. On average, NB, KNN, and ANNs have 88.40%, 96.80%, and 97.20% recognition rates, respectively. In the SU-AIS BB-MAS dataset, it is easier to detect walking activity than the other two activities. We also find it is harder to identify going downstairs than going upstairs. Compared to our own dataset of eight users, the experimental results of SU-AIS BB-MAS show that the accuracy of the three classifiers decreases slightly. Although ANNs provides the best recognition rate among the three classifiers, the number of hidden layer nodes increases from 8 to 64. The reason is that the gait data of our dataset are collected from a smartphone fixed on the user’ right thigh while the raw data of SU-AIS BB-MAS are obtained from a smartphone placed in the participant’s pocket arbitrarily.

### 4.4. Live Detection

**Figure 5:**
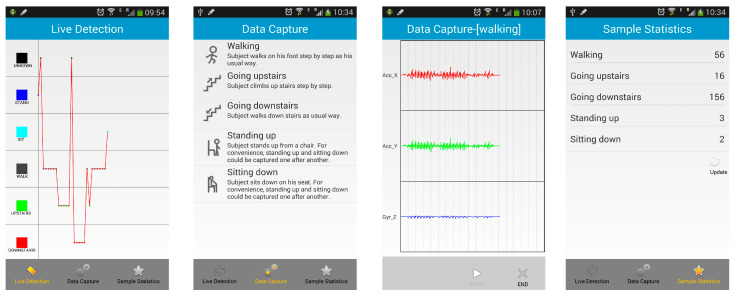
Screenshots of Live Detection System.

To validate the availability of our motion recognition approach, we design and implement a human lower limb motion live detection program, which includes three main modules: data capture, sample statistics, and live detection, as shown in Figure 5. We develop this live detection program for Android-based Smartphones and run it on Samsung Galaxy Note 3. This application is designed based on an offline training and online prediction scheme. This means the ANNs model training and the obtaining of optimal parameters are performed on a remote computer, whereas human motion recognition is performed on a battery-driven smartphone in order to reduce the battery dissipation and enhance the real-time response. The end user can capture the raw sensing data of their five activities when the subject does the corresponding physical activity they select in the data capture module. After finishing the data capture, the subject can obtain the samples of each activity utilizing the sample statistics module and feed these samples into the classifier to train the recognition model in the remote server for saving battery energy. The subject’s five lower limb activities can then be recognized in real time using the live detection module. In the live detection module, we use our proposed live detection algorithm to recognize the mobile user’s lower limb motion in real time.

The live detection system provides users a report of their daily lower limb physical activity in real time. The activity category of each activity is displayed on the mobile device as shown in Figure 5. The value of the application is that it can demonstrate that our proposed approach can recognize human lower limb motion with a high accuracy, and it also provides a chance to detect abnormal movement in the future work of this study, such as automated fall detection.

**Figure 6:**
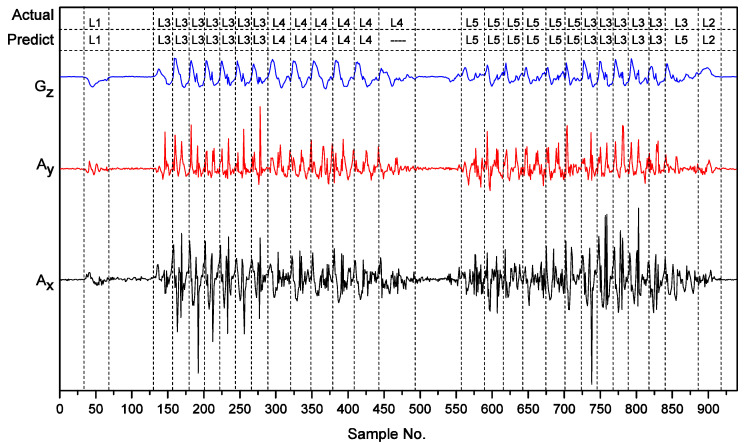
Live detection results of a subject’s serial activities.

We record the sensing data of the accelerometer and gyroscope while subjects do the following serial activities, including 939 sample points as shown in Figure 6:

1. sitting in a chair for 2090 milliseconds;
2. standing up from a chair;
3. standing still for 3756 milliseconds;
4. walking forward seven steps;
5. climbing upstairs, and after six steps, turning around;
6. standing still for 2330 milliseconds;
7. walking downstairs;
8. walking toward to the chair six steps and then turning around;
9. sitting down on the seat;
10. sitting in a chair for 1850 milliseconds.

From Figure 6, we can see that our live detection algorithm can extract each human activity cycle from the time serial accurately. After feeding each activity cycle into our human lower limb motion recognition approach, we achieve a high recognition result. Our approach recognizes 25 motions among 27 recognizable activities, except standing still and turning around. However, we cannot identify the last step of climbing stairs and improperly classify a walking activity as walking downstairs because we cannot separate the turning around cycle properly.

## 5. Conclusions

This paper proposes a human lower limb motion capturing and recognition method using the sensors built into any Android-based Smartphone, respecting the benefits and pitfalls of existing motion capture systems. By placing the Smartphone on the upper leg, one avoids any expensive, e.g., wearable, or pervasive systems. We can reliably recognize the following five lower limb motions: standing up, sitting down, walking, going upstairs, and going downstairs. We use FFT extracting features in the frequency domain of motion sensing data and reduce the feature vector size to decrease the complexity of the recognition system. Furthermore, we use the 10-fold cross-validation technique to evaluate the three classifiers. Finally, we designed and implemented a live detection application using a real-time detection algorithm based on our motion approach to prove that our lower limb motion recognition method can achieve very high accuracy. We did not consider the abnormal movement detection problem in this paper. In the future, we plan to improve the recognition rate for going upstairs and going downstairs and reconstruct the human lower limb motion in real time using the public dataset SU-AIS BB-MAS. We also would like to extend this application further to support more lower limb motion activities in order to detect mobile users’ abnormal movement for some special groups, such as medical patients and elderly people.

## Figures and Tables

**Table 1:** The diversity of motion samples.

Human Lower Limb Motion	Variety of Test Condition	Number	Percentage
Walking	Stride length: 50~90 cm	156	23%
Going upstairs	Step height: 17 cm, 20 cm	122	18%
Going downstairs	Step height: 17 cm, 20 cm	113	17%
Standing up	Seat height: 32 cm, 42 cm, 48 cm	139	21%
Sitting down	Seat height: 32 cm, 42 cm, 48 cm	140	21%

**Table 2:** Human limb motion recognition results of three classifiers.

Classifier	Accuracy	Limb Motion	TPR	FPR	Precision	Recall
NB (*n* = 6)	97.01%	Standing up	97.75%	0.19%	99.23%	97.75%
		Sitting down	100%	0%	100%	100%
		Walking	93.00%	0.57%	97.91%	93.00%
		Going upstairs	96.86%	1.29%	94.42%	96.86%
		Going downstairs	98.44%	1.64%	92.85%	98.44%
		Average	97.21%	0.74%	96.88%	97.21%
KNN (*n* = 6, *K* = 1)	96.12%	Standing up	97.64%	0.19%	99.33%	97.64%
		Sitting down	100%	0%	100%	100%
		Walking	94.43%	1.77%	94.24%	94.43%
		Going upstairs	93.79%	2.68%	87.67%	93.79%
		Going downstairs	93.37%	0.16%	98.57%	93.37%
		Average	95.85%	0.96%	95.96%	95.85%
ANNs (*n* = 6, *numHLN* = 8)	98.21%	Standing up	99.41%	0.21%	99.50%	99.41%
		Sitting down	100%	0.19%	99.29%	100%
		Walking	99.41%	0.98%	96.86%	99.41%
		Going upstairs	97.98%	0.71%	96.42%	97.98%
		Going downstairs	93.34%	0.18%	99.00%	93.34%
		Average	98.03%	0.45%	98.21%	98.03%

**Table 3:** Confusion matrix for five human activities.

Actual	NB Predicted Results					KNN Predicted Results					ANN Predicted Results				
	L1	L2	L3	L4	L5	L1	L2	L3	L4	L5	L1	L2	L3	L4	L5
L1	136	0	0	1	2	136	0	1	2	0	138	0	0	1	0
L2	0	140	0	0	0	0	140	0	0	0	0	140	0	0	0
L3	0	0	145	4	7	0	0	147	9	0	0	0	155	0	1
L4	1	0	3	118	0	1	0	5	115	1	2	0	0	120	0
L5	0	0	0	2	111	0	0	3	4	106	0	0	5	3	105

**Table 4:** Activity recognition results of three classifiers using SU-AIS BB-MAS.

Classifier	Accuracy	Limb Motion	TPR	FPR	Precision	Recall
NB (*n* = 6)	88.40%	Walking	100.00%	0.33%	99.78%	100.00%
		Going upstairs	82.76%	9.87%	72.58%	82.28%
		Going downstairs	67.57%	5.25%	81.51%	67.57%
		Average	83.44%	5.15%	84.62%	83.44%
KNN (*n* = 6, *K* = 1)	96.80%	Walking	100.00%	0.28%	99.75%	100.00%
		Going upstairs	93.91%	2.27%	92.66%	93.91%
		Going downstairs	92.43%	1.68%	93.46%	92.43%
		Average	95.44%	1.41%	95.29%	95.44%
ANNs (*n* = 32, *numHLN* = 64)	97.20%	Walking	100.00%	0.00%	100.00%	100.00%
		Going upstairs	94.52%	1.76%	94.43%	94.52%
		Going downstairs	93.75%	1.86%	93.24%	93.75%
		Average	96.09%	1.21%	95.89%	96.09%

## Data Availability

Not applicable.

## References

[1] Chen X., Tan T., Cao G., Porta T.F.L. (2022). Context-Aware and Energy-Aware Video Streaming on Smartphones. IEEE Trans. Mob. Comput..

[2] Chon J., Cha H. (2011). LifeMap: A Smartphone-Based Context Provider for Location-Based Services. IEEE Pervasive Comput..

[3] Sysoev M., Kos A., Pogačnik M. (2015). Noninvasive Stress Recognition Considering the Current Activity. Pers. Ubiquit. Comput..

[4] Cornacchia M., Ozcan K., Zheng Y., Velipasalar S. (2017). A Survey on Activity Detection and Classification Using Wearable Sensors. IEEE Sens. J..

[5] Khimraj P.K., Shukla K.P., Vijayvargiya A., Kumar R. Human Activity Recognition Using Accelerometer and Gyroscope Data from Smartphones. Proceedings of the International Conference on Emerging Trends in Communication, Control and Computing.

[6] Testoni A., Di Felice M. A Software Architecture for Generic Human Activity Recognition from Smartphone Sensor Data. Proceedings of the IEEE International Workshop on Measurement and Networking.

[7] Vyas R., Doddabasappla K. (2022). FFT Spectrum Spread with Machine Learning (ML) Analysis of Triaxial Acceleration from Shirt Pocket and Torso for Sensing Coughs While Walking. IEEE Sens. Lett..

[8] Nguyen H., Lebel K., Bogard S., Goubault E., Boissy P., Duval C. (2018). Using Inertial Sensors to Automatically Detect and Segment Activities of Daily Living in People with Parkinson’s Disease. IEEE Trans. Neural Syst. Rehabil. Eng..

[9] Wang M., Luo C., Ni B., Yuan J., Wang J., Yan S. (2018). First-Person Daily Activity Recognition with Manipulated Object Proposals and Non-Linear Feature Fusion. IEEE Trans. Circuits Syst. Video Technol..

[10] Hamdi M.M., Awad M., Abdelhameed M.M., Tolbah F. Lower Limb Motion Tracking Using IMU Sensor Network. Proceedings of the Cairo International Biomedical Engineering Conference.

[11] Chinimilli P.T., Redkar S., Sugar T. (2019). A Two-Dimensional Feature Space-Based Approach for Human Locomotion Recognition. IEEE Sens. J..

[12] Filios G., Nikoletseas S., Pavlopoulou C. Efficient Parameterized Methods for Physical Activity Detection Using Only Smartphone Sensors. Proceedings of the 13th ACM International Symposium on Mobility Management and Wireless Access.

[13] Anjum A., Ilyas M.U. Activity Recognition Using Smartphone Sensors. Proceedings of the Consumer Communications and Networking Conference.

[14] Belman A.K., Wang L., Iyengar S.S., Sniatala P., Wright R., Dora R., Baldwin J., Jin Z.P., Phoha V.V. (2019). Insights from BB-MAS-A Large Dataset for Typing, Gait and Swipes of the Same Person on Desktop, Tablet and Phone. arXiv.

[15] Belman A.K., Wang L., Iyengar S.S., Sniatala P., Wright R., Dora R., Baldwin J., Jin Z.P., Phoha V.V. (2019). SU-AIS BB-MAS (Syracuse University and Assured Information Security—Behavioral Biometrics Multi-device and multi-Activity data from Same users) Dataset. IEEE Dataport.

[16] Maurer U., Smailagic A., Siewiorek D.P., Deisher M. Activity Recognition and Monitoring Using Multiple Sensors on Different Body Positions. Proceedings of the International Workshop on Wearable and Implantable Body Sensor Networks.

[17] Ma H., Liu H. Research on Human Motion Recognition System Based on MEMS Sensor Network. Proceedings of the 4th Advanced Information Technology, Electronic and Automation Control Conference.

[18] Ermes M., Parkka J., Mantyjarvi J., Korhonen I. (2008). Detection of Daily Activities and Sports with Wearable Sensors in Controlled and Uncontrolled Conditions. IEEE Trans. Inf. Technol. Biomed..

[19] Hussain F., Hussain F., Ehatisham-ul-Haq M., Azam M.A. (2019). Activity-Aware Fall Detection and Recognition Based on Wearable Sensors. IEEE Sens. J..

[20] Jain A., Kanhangad V. (2018). Human Activity Classification in Smartphones Using Accelerometer and Gyroscope Sensors. IEEE Sens. J..

[21] Besler E., Mathur P.K., Gay H.C., Passman R.S., Sahakian A.V. (2022). Inter-Patient Atrial Flutter Classification Using FFT-Based Features and a Low-Variance Stacking Classifier. IEEE Trans. Biomed. Eng..

[22] Wang A., Chen G., Jing Y., Zhao S., Chang C.Y. (2016). A Comparative Study on Human Activity Recognition Using Inertial Sensors in a Smartphone. IEEE Sens. J..

